# A versatile “3M” methodology to obtain superhydrophobic PDMS-based materials for antifouling applications

**DOI:** 10.3389/fbioe.2022.998852

**Published:** 2022-08-29

**Authors:** Zhoukun He, Xiaochen Yang, Linpeng Mu, Na Wang, Xiaorong Lan

**Affiliations:** ^1^ Institute for Advanced Study, Research Center of Composites and Surface and Interface Engineering, Chengdu University, Chengdu, China; ^2^ School of Mechanical Engineering, Chengdu University, Chengdu, China; ^3^ Luzhou Key Laboratory of Oral & Maxillofacial Reconstruction and Regeneration, The Affiliated Stomatological Hospital of Southwest Medical University, Luzhou, China; ^4^ Institute of Stomatology, Southwest Medical University, Luzhou, China

**Keywords:** superhydrophobic, PDMS, nanoparticles, aggregates, antifouling

## Abstract

Fouling, including inorganic, organic, bio-, and composite fouling seriously affects our daily life. To reduce these effects, antifouling strategies including fouling resistance, release, and degrading, have been proposed. Superhydrophobicity, the most widely used characteristic for antifouling that relies on surface wettability, can provide surfaces with antifouling abilities owing to its fouling resistance and/or release effects. PDMS shows valuable and wide applications in many fields, and due to the inherent hydrophobicity, superhydrophobicity can be achieved simply by roughening the surface of pure PDMS or its composites. In this review, we propose a versatile “3M” methodology (materials, methods, and morphologies) to guide the fabrication of superhydrophobic PDMS-based materials for antifouling applications. Regarding materials, pure PDMS, PDMS with nanoparticles, and PDMS with other materials were introduced. The available methods are discussed based on the different materials. Materials based on PDMS with nanoparticles (zero-, one-, two-, and three-dimensional nanoparticles) are discussed systematically as typical examples with different morphologies. Carefully selected materials, methods, and morphologies were reviewed in this paper, which is expected to be a helpful reference for future research on superhydrophobic PDMS-based materials for antifouling applications.

## Introduction

There are four main types of fouling according to the nature of the foulant, namely, inorganic, organic, bio-, and composite fouling (He et al., 2021a; He et al., 2021b). Fouling seriously affects daily life. For example, in biofouling (Callow and Callow, 2011; Bixler et al., 2014; Manolakis and Azhar, 2020), various unwanted organisms attach to the surfaces of metallic, ceramic, polymeric, or composite products; this leads to increased fuel consumption and corrosion in marine biofouling (Dobretsov et al., 2013; Mieszkin et al., 2013; Gaw et al., 2017; Hu et al., 2020), hospital-acquired infections in medical biofouling (Jorge et al., 2012; Ammons and Copié, 2013; Leslie et al., 2014), or function decline in industrial biofouling (Bixler and Bhushan, 2012; Liang et al., 2020; Obotey Ezugbe and Rathilal, 2020). Various antifouling strategies involving fouling resistance, release, and degrading, have been proposed (Zhao et al., 2018; Maan et al., 2020). Inspired by nature, such as the anti-wettability of lotus leaf, rice leaf, and shark skin effects, scientists have developed many well-known bionic antifouling coatings with different surface wettability properties (Ball, 1999; Roach et al., 2008; Zhu et al., 2010; Scardino and de Nys, 2011; Wu et al., 2011; Bixler and Bhushan, 2013; Bixler and Bhushan, 2014; Azemar et al., 2015; Jiang et al., 2015; Zhang et al., 2016; Pan et al., 2019; Zarghami et al., 2019; Basu et al., 2020).

**FIGURE 1:**
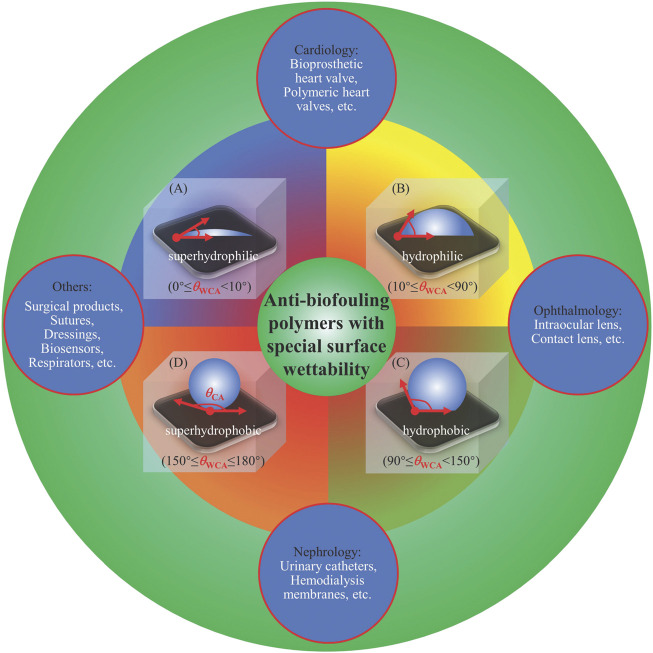
Anti-biofouling polymers with different surface wettability for various biomedical applications. Reprinted with permission from Ref. (He et al., 2021a).

In our previous publications, we discussed the relationship between antifouling and surface wettability (He et al., 2021a; He et al., 2021b; Lan et al., 2021; Lei et al., 2021). For example, we summarized the frequent strategies to achieve anti-biofouling polymers for biomedical applications based on different types of surface wettability (He et al., 2021a), including superhydrophilicity (Figure 1A), hydrophilicity (Figure 1B), hydrophobicity (Figure 1C), and superhydrophobicity (Figure 1D). Examples with suitable polymers were introduced for specific applications *in vivo* and *in vitro*, such as cardiological (bioprosthetic heart valves, polymeric heart valves, etc.), ophthalmological (intraocular lenses, contact lenses, etc.), nephrological (urinary catheters, hemodialysis membranes, etc.), and other applications (surgical products, sutures, dressings, biosensors, respirators, etc.).

**FIGURE 2:**
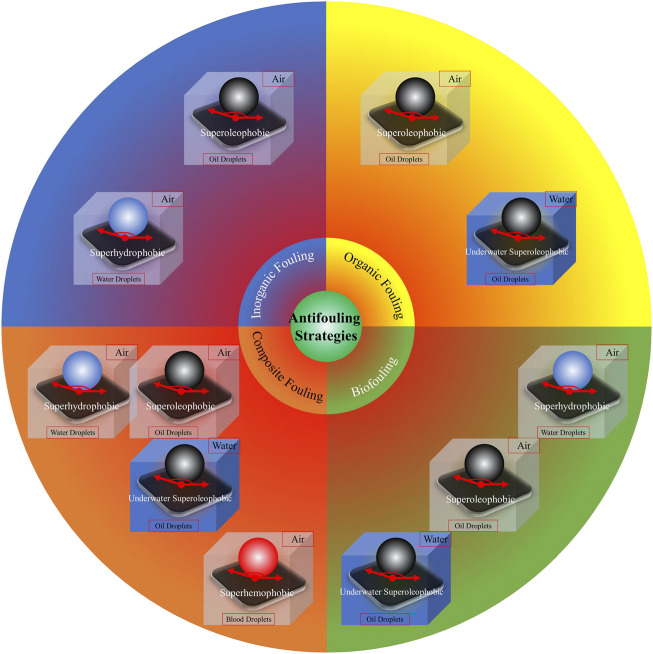
Antifouling strategies based on super-phobic surfaces. Reprinted with permission from Ref. (He et al., 2021b). Copyright 2021, Elsevier B.V.

Among the four mentioned types of surface wettability, superhydrophobicity (which is the most common research focus in the field) can confer antifouling abilities to various surfaces owing to its fouling-resistant and/or fouling-release properties (Wang and Jiang, 2007; Xia and Jiang, 2008; Ma et al., 2015; Liu et al., 2020a; Simovich et al., 2020). Similarly, other kinds of super-phobicity, such as superoleophobicity, underwater superoleophobicity, and superhemophobicity, can lead to adequate antifouling effects owing to a decreasing adhesion strength between the foulant and substrate (He et al., 2021b). Notably, different types of super-phobicity could be effective against different types of fouling (Figure 2). Superhydrophobicity and superoleophobicity are the most applied super-phobicity strategies in antifouling fields.

It is known that surface wettability is a result of the surface chemical composition and physical structure (Young, 1805; Zhu et al., 2012; Tian et al., 2014; Yu et al., 2015; Kuang et al., 2016; Martin et al., 2017; Lee et al., 2022; Luo et al., 2022; Zhang et al., 2022). Silicone- or fluoro-based polymers are the main polymeric materials used to achieve superhydrophobicity or superoleophobicity (Dobretsov and Thomason, 2011), and silicone- and fluoro-based polymers with fouling release properties are suitable for achieving antifouling abilities (Carl et al., 2012; Lejars et al., 2012; Liu et al., 2017; Liang et al., 2020). However, fluoro-based materials are expensive and may result in irreversible pollution due to fluoride toxicity (Cao et al., 2022). By contrast, silicone-based polymers, such as polydimethylsiloxane (PDMS), are advantageous owing to their acceptable costs, chemical stability, biocompatibility, and weatherability (Liu et al., 2021). There are 1,025 publications related to superhydrophobic PDMS (Supplementary Figure S1, searched in all fields in Web of Science with “superhydrophobic” and “PDMS” on July 14th, 2022), but only 7 review papers based on this topic are searchable (Supplementary Figure S2). After checked these 7 review papers one by one, there is no review paper focused on the antifouling applications based on superhydrophobic PDMS materials. Therefore, it is necessary to summarize this topic in order to provide a helpful reference for future research on superhydrophobic PDMS-based materials for antifouling applications.

## “3M” methodology to obtain superhydrophobic polydimethylsiloxane-based materials

### “3M” methodology

**FIGURE 3:**
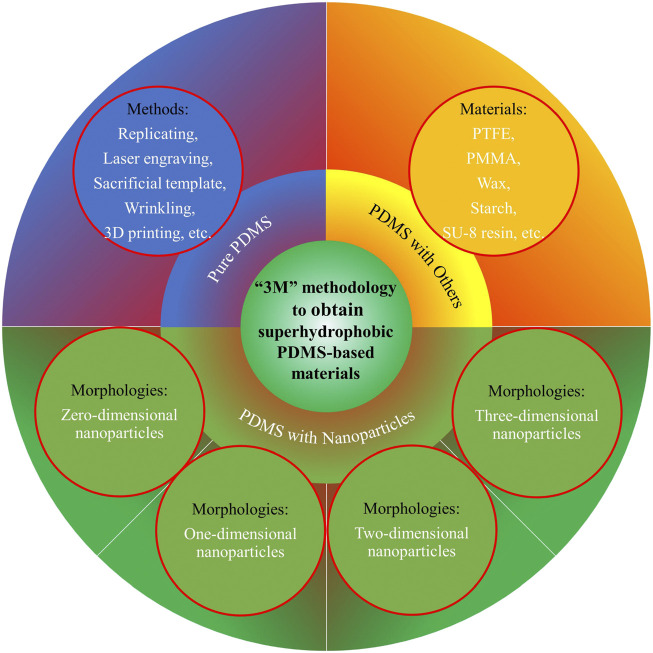
A versatile “3M” (materials, methods, and morphologies) methodology to obtain superhydrophobicity on PDMS-based materials.

A versatile “3M” (materials, methods, and morphologies) methodology to obtain superhydrophobicity easily and universally is proposed in this review as a guide for future research. The “3M” methodology (Figure 3) underlies the strategies for obtaining all types of PDMS-based superhydrophobic materials (pure PDMS, PDMS with nanoparticles, and PDMS with other materials), although each type has its own focus and character. For example, for pure PDMS-based superhydrophobicity, the material is PDMS, but the chosen fabrication method must consider the expected final morphology. Similarly, for PDMS with nanoparticles-based superhydrophobicity, the nanoparticle morphology together with its specific material, and the fabrication method of PDMS with nanoparticles should be considered simultaneously. The “3M” methodology also works for the third superhydrophobicity type (based on PDMS with other materials). Thus, the proposed “3M” methodology can be summarized in the following sentence: “The use of specific materials and methods to construct special morphologies for surface superhydrophobicity;” thus, it can be extrapolated to various fields that require surfaces with superhydrophobicity or other special surface wettability properties.

### Superhydrophobicity based on different polydimethylsiloxane materials

PDMS is an optically clear, inert, nontoxic material that is widely applied in medical devices, cosmetics, elastomers, antifoaming agents, flexible sensors, stretchable electronics, and other valuable domestic applications (Das et al., 2018; Zaman et al., 2019; Wang et al., 2021a; Liu et al., 2021; Qi et al., 2021). Due to the inherent hydrophobicity of PDMS, superhydrophobicity can be achieved simply by roughening the surface of pure PDMS or its composites (Figure 3).

Pure PDMS can be roughened to obtain superhydrophobicity *via* replication (Liu et al., 2006; Cho and Choi, 2008; Park et al., 2011; Dai et al., 2019; Liu et al., 2019; Schultz et al., 2020; Siddiquie et al., 2020), laser engraving (Yong et al., 2013; Yong et al., 2017; Zhao et al., 2019a; Zhang et al., 2020; Chen et al., 2021), introducing a sacrificial template (Yu et al., 2017; Davis et al., 2018), wrinkling (Zhao et al., 2013), 3D printing (He et al., 2017; Chen et al., 2019), and other methods (Zimmermann et al., 2008a; Zimmermann et al., 2008b; Artus and Seeger, 2014; Seo et al., 2016; Wang et al., 2021b; Mazaltarim et al., 2021; Siddiqui et al., 2021; Park et al., 2022). As an example, for the PDMS to achieve superhydrophobicity, the replication methods use different molds, including natural morphologies (lotus leaves (Liu et al., 2006), rose petals (Dai et al., 2019), shark skin surfaces (Liu et al., 2019), etc.), and artificial morphologies (periodic or multiscale structures produced *via* femtosecond laser processing on stainless-steel substrates (Siddiquie et al., 2020), cylindrical silicon trenches produced *via* reactive ion etching (Park et al., 2011), polycarbonate spherulite networks produced *via* a controlled solvent treatment (Schultz et al., 2020), nanoporous anodic aluminum oxides produced *via* two-step anodization (Cho and Choi, 2008), etc.). Although the methods or morphologies may differ, the purpose is to introduce micro- and nano-scale or hierarchical roughness into the hydrophobic PDMS material to obtain superhydrophobicity.

The case of materials based on PDMS with nanoparticles is different from that of pure PDMS materials because nanoparticles possess inherently rough structures that can be directly exploited to fabricate PDMS-based superhydrophobic surfaces. Nanoparticle materials can be classified by morphology into four types: zero-dimensional nanoparticles (He et al., 2011; He et al., 2012; Zhao et al., 2015; Aslanidou et al., 2016; Selim et al., 2018a; Davis et al., 2018; Liu et al., 2018; Saharudin et al., 2018; Lu et al., 2019; Liu et al., 2020b; Gu et al., 2020; Han and Gong, 2021; Xiong et al., 2022a; Rin Yu et al., 2022; Yu et al., 2022) (such as spherical silicon dioxide (SiO_2_) (Aslanidou et al., 2016; Yu et al., 2022), titanium dioxide (TiO_2_) (Liu et al., 2020b), polypyrrole nanoparticles (Xiong et al., 2022a), core-shell spherical composite nanoparticles (Selim et al., 2018a), or hollow spherical nanoclusters (Han and Gong, 2021)), one-dimensional nanoparticles (Wang et al., 2019a; Dai et al., 2019; Selim et al., 2019; Li et al., 2021) (such as linear nanorods (Selim et al., 2019) and carbon nanotubes (CNTs) (Li et al., 2021)), two-dimensional nanoparticles (Wang et al., 2019b; Saharudin et al., 2019; Li and Guo, 2020; Cao et al., 2021) (such as laminar graphene and its derivatives (Li and Guo, 2020), iron oxide (Fe_3_O_4_) nanoplates (Cao et al., 2021)), and three-dimensional nanoparticles (single material nanoparticles such as tetrapod-shaped zinc oxide (ZnO) (Yamauchi et al., 2019) and flower-like calcium titanium (CaTiO_3_) structures (Wang et al., 2007), and composite nanoparticles (Nine et al., 2015; Shi et al., 2015; Barthwal et al., 2020; Zhu et al., 2020; Zhang et al., 2021a; Zhang et al., 2021b; Wu et al., 2021; Selim et al., 2022a; Selim et al., 2022b; Xiong et al., 2022b; Cheng et al., 2022; Miao et al., 2022), among which are dual-sized SiO_2_ with micropowder and nanofumed morphologies (Zhang et al., 2021a), polydopamine clusters integrated with SiO_2_ to create micro-nano composite structures (Miao et al., 2022), TiO_2_ and SiO_2_ composite nanoparticles (Xiong et al., 2022b), and CNT and Fe_3_O_4_ composites (Wu et al., 2021)). For example, SiO_2_ nanoparticles themselves have nano-scale roughness and the aggregates formed by the particles provide additional hierarchical roughness (He et al., 2011; Gao and Yan, 2012; He et al., 2012; He et al., 2013; Yu et al., 2018). Therefore, materials based on PDMS and nanoparticles easily satisfy the roughness requirements for superhydrophobicity.

For PDMS with other materials, various methods can be used to obtain superhydrophobicity, such as spin coating with PDMS and polytetrafluoroethylene (PTFE) powder (Ruan et al., 2017), electrospinning to produce PDMS and poly (methyl methacrylate) (PMMA) composites (Lu et al., 2021), drop casting or spray coating with PDMS and wax (Zhao et al., 2019b; Torun et al., 2019; Celik et al., 2021), spray coating with PDMS and starch (Wang et al., 2021c), preparing PDMS films with SU-8 resin (Wu et al., 2018), and other methods (Děkanovský et al., 2019; Pakzad et al., 2020; Cao et al., 2022; Zhao et al., 2022). The combined action of the used materials and methods leads to specific morphologies that result in superhydrophobicity.

The comparison of different typical superhydrophobic PDMS-based materials is listed in Table 1. For different types of superhydrophobic PDMS-based materials, materials, methods, and morphologies are summarized and sorted to compare with each other.

**TABLE 1:** Comparison of different typical superhydrophobic PDMS-based materials.

Categories	Materials		Methods		Morphologies		Ref.
Pure PDMS	PDMS		Soft-Lithographic Imprinting	Template: lotus leaves, rose petal, shark skin	Natural	Lotus-leaf-like, rose-petal-like, shark-skin-like surfaces	Liu et al. (2006); Dai et al. (2019); Liu et al. (2019)
				Template: stainless-steel roughened by femtosecond laser	Artificial	Periodic or multiscale structures	Siddiquie et al. (2020)
				Template: cylindrical silicon trenches produced by reactive ion etching		Ordered microshell array	Park et al. (2011)
				Template: polycarbonate spherulite networks produced *via* a controlled solvent treatment		Negative spherulite networks	Schultz et al. (2020)
				Template: nanoporous anodic aluminum oxides produced *via* two-step anodization		Hairy nanopillar	Cho and Choi. (2008)
			Laser engraving	D80M multi-function laser engraving machine	Artificial	Various columns, holes, grooves	Zhao et al. (2019a)
				Nanosecond fiber laser (SPI, 74W EP-Z): a wavelength of 1064 nm and a pulse width of 120 ns		Expanded cracks and holes	Chen et al. (2021)
				Femtosecond laser ablation: wavelength, duration, and repetition rate of the laser beam were 800 nm, 50 fs, and 1 kHz, respectively		Micro-/nanoscale hierarchical rough structures	Yong et al. (2017)
				Nanosecond UV laser (Nd: YVO4)		Grooves	Zhang et al. (2020)
				Femtosecond Laser: wavelength of 800 nm with a repetition rate of 1 kHz		Square array pattern	Yong et al. (2013)
			Sacrificial template	Salt, sugar, water, etc.	Artificial	Porous sponge	Yu et al. (2017); Davis et al. (2018)
			Wrinkling	Mechanical stretch	Artificial	Grooves	Zhao et al. (2013)
			3D printing	Direct ink writing	Artificial	Porous	He et al. (2017); Chen et al. (2019)
			Polymerization	Ultrasonication-induced and diluent-assisted suspension polymerization	Natural	Rose-petal-like monodisperse droplets	Park et al. (2022)
			Polymerization	Gas phase polymerization	Artificial	Nanofilaments	Zimmermann et al. (2008a); Zimmermann et al. (2008b)
PDMS with nanoparticles	PDMS, zero-dimensional nanoparticles	Spherical SiO_2_	Coating	Spin, dip, spray coating, casting, etc.	Spontaneous	Nanoparticle aggregates	He et al. (2011); He et al. (2012); Aslanidou et al. (2016); Liu et al. (2018); Saharudin et al. (2018); Lu et al. (2019)
		Spherical TiO_2_	Coating	Dip coating	Spontaneous	Nanoparticle aggregates	Zhao et al., (2015); Liu et al., (2020b)
		Spherical Ag@ SiO_2_ core-shell nanocomposite	Coating	Casting	Spontaneous	Nanoparticle aggregates	Selim et al. (2018a)
	PDMS, one-dimensional nanoparticles	Linear ZnO nanorods	Coating	Casting, brush coating, etc.	Spontaneous	Nanoparticle aggregates	Selim et al. (2019)
		Linear ZnO nanorods	Hydrothermal reaction	Growing with ZnO seed	Spontaneous	Nanoparticle aggregates	Dai et al. (2019)
		CNTs	Coating	Spray coating, casting	Spontaneous	Nanoparticle aggregates	Wang et al. (2019a); Li et al. (2021)
	PDMS, two-dimensional nanoparticles	Laminar graphene	Coating	Spray coating	Spontaneous	Nanoparticle aggregates	Saharudin et al. (2019)
		Laminar graphene	Coating	Blade coating	Spontaneous	Nanoparticle aggregates	Wang et al. (2019b)
		Laminar nano-graphite flakes	Coating	Dip coating	Spontaneous	Nanoparticle aggregates	Li and Guo, (2020)
	PDMS, three-dimensional nanoparticles	Tetrapod-shaped ZnO	Coating	Spray coating	Natural	Porcupinefish-like aggregates	Yamauchi et al. (2019)
		Flower-like CaTiO_3_ structures	Hydrothermal reaction	Etching of titanium by a base solution and instant growth	Natural	Flower-like aggregates	Wang et al. (2007)
		Dual-sized sphericalSiO_2_ with micropowder and nanofumed morphologies	Coating	Spray coating	Spontaneous	Nanoparticle aggregates	Zhang et al. (2021a)
		Dual-sized sphericalSiO_2_ nanoparticles with spherical pigment	Coating	Brush coating	Natural	Raspberry-like aggregates	Cheng et al. (2022)
		Dual-sized linear multi-walled CNTs and spherical ZnO composite	Coating	Dip coating	Spontaneous	Nanoparticle aggregates	Barthwal et al. (2020)
		Dual-sized laminar graphene oxide (GO) and linear TiO_2_ nanorods	Coating	Brush coating	Spontaneous	Nanoparticle aggregates	Selim et al. (2022a)
PDMS with others	PDMS, PTFE powder		Coating	Spin coating	Natural	Honeycomb-like structures	Ruan et al. (2017)
	PDMS, PMMA		Electrospinning		Spontaneous	Porous membrane with bead on string	Lu et al. (2021)
	PDMS, carnauba wax		Coating	Spray coating, casting	Natural	Lotus-leaf-like structures	Torun et al. (2019); Celik et al. (2021)
	PDMS, paraffin wax		Coating	Dip coating	Spontaneous	Randomly scattered structures	Zhao et al. (2019b)
	PDMS, starch		Coating	Spray coating	Spontaneous	Hierarchical structures	Wang et al. (2021c)

Among these three types of superhydrophobic materials based on PDMS, the PDMS with nanoparticles type has many advantages with respect to the other two types. First, the nanoparticles with different morphologies can be obtained easily and inexpensively and may confer other functional properties to the materials, such as photocatalytic (Liu et al., 2020b; Chen et al., 2022), electrical conductivity (Wang et al., 2019c; Li and Guo, 2020), thermochromic (Cheng et al., 2022), and self-illuminous properties (Shi et al., 2015). Second, the methods to achieve superhydrophobicity with PDMS with nanoparticles are based on one-step coating strategies (Steele et al., 2009), which are easier and less costly than strategies such as replicating, laser engraving, and electrospinning. Third, hierarchical roughness can be obtained *via* the spontaneous formation of nanoparticle aggregates (He et al., 2011; Gao and Yan, 2012; He et al., 2012; He et al., 2013; Yu et al., 2018). Here, we will use materials based on PDMS with nanoparticles to exemplify the “3M” methodology in the following sections.

## Superhydrophobicity based on polydimethylsiloxane with nanoparticle aggregates

**FIGURE 4:**
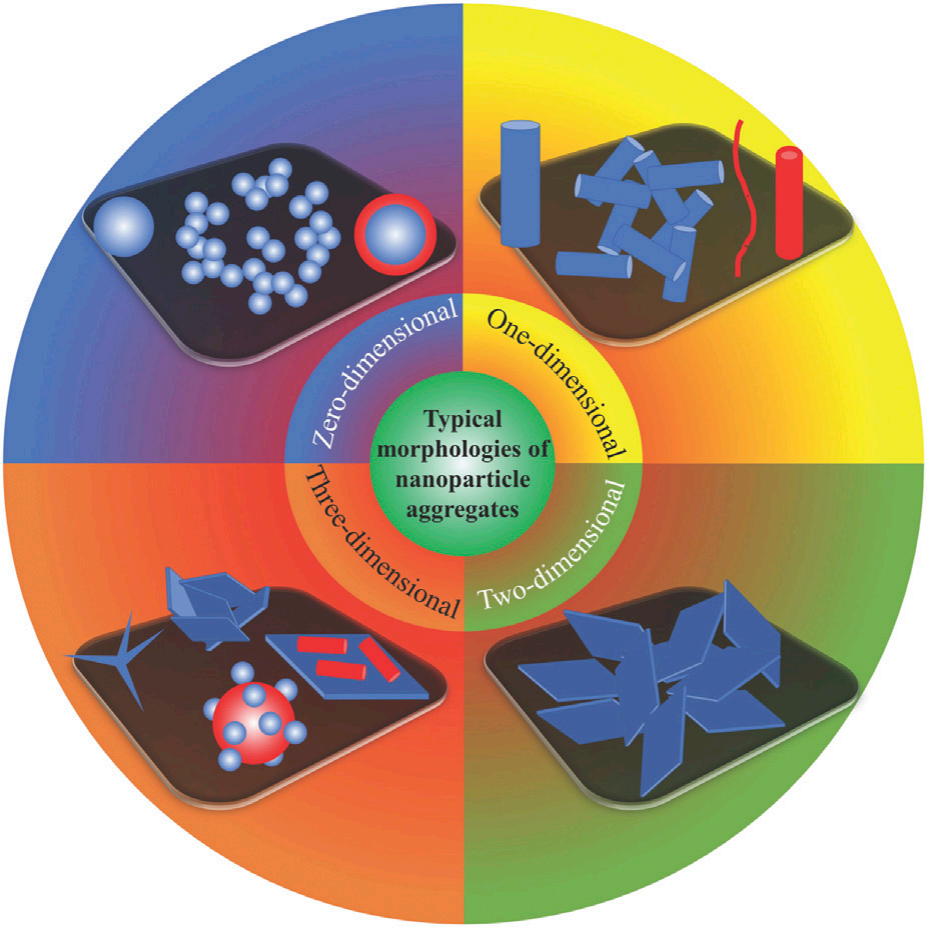
Typical nanoparticle aggregate morphologies.

Nanoparticles with different morphologies form different aggregates; the typical aggregate morphologies are shown in Figure 4. Zero-dimensional nanoparticles may be made of a single, two, or more types of materials with core–shell structures. One-dimensional nanoparticles can have many different morphologies, such as nanorods, nanowires and nanotubes. The morphologies of two-dimensional nanoparticles are usually simple laminar or layered structures. Three-dimensional nanoparticles can consist of single materials (such as the shown tetrapod-shaped or flower-like particles) or composite nanoparticles. The latter can be combinations of differently sized nanoparticles with the same dimensional morphology (for example, raspberry-like structures) or combinations of nanoparticles with different dimensional morphologies (for example, linear one-dimensional nanoparticles on the surface of laminar two-dimensional nanoparticles). Regardless of their specific morphology, nanoparticles aggregate spontaneously to form various hierarchical structures. Usually, aggregates of PDMS with nanoparticles are similar in morphology to those without PDMS. In this context, the nanoparticle aggregates usually provide the necessary hierarchical roughness to achieve superhydrophobicity and the PDMS provides a low surface energy and binds the aggregates together.

### Polydimethylsiloxane with zero-dimensional nanoparticles

**FIGURE 5:**
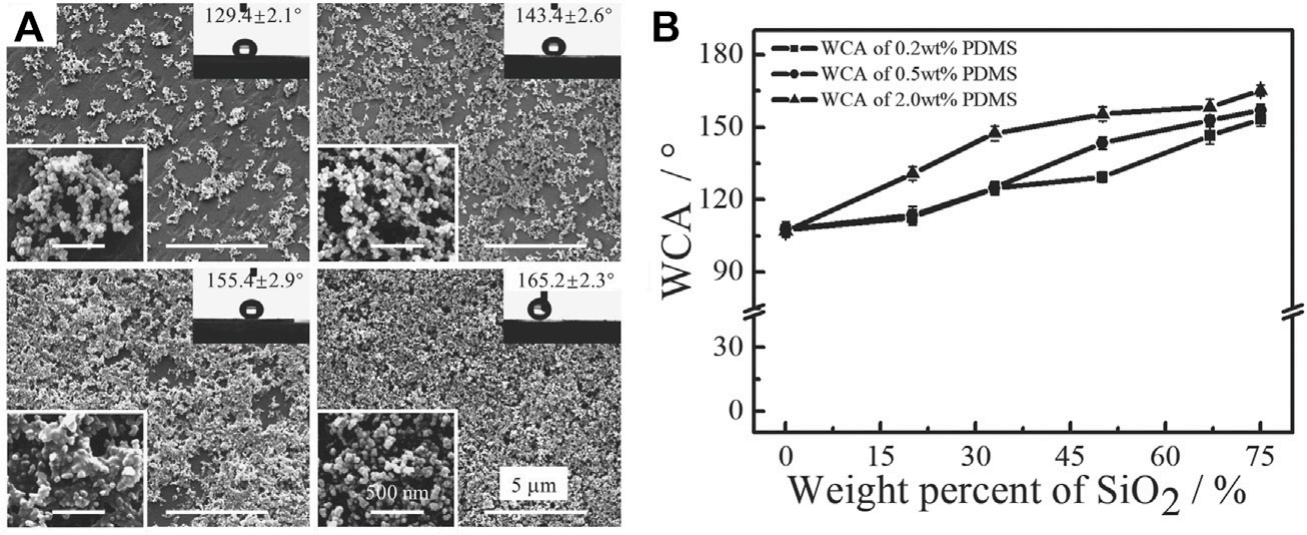
Superhydrophobic coatings fabricated with PDMS and SiO_2_ nanoparticles. SEM images of the physical morphologies **(A)**, and WCA changes with the weight percent of nanoparticles **(B)**. Reprinted with permission from Ref. (He et al., 2012). Copyright 2012, Elsevier B.V.

A facile and universal strategy to fabricate superhydrophobic surfaces *via* spin-coating a mixture of PDMS and SiO_2_ nanoparticles on a target substrate was proposed in our previous publications (He et al., 2011; He et al., 2012). As shown in Figure 5A, multi-scale physical structures with micro-scale nanoparticle aggregates and nano-scale single nanoparticles were obtained *via* one-step coating; this was attributed to spontaneous nanoparticle aggregates (Liu et al., 2008; Wang et al., 2008; Xu et al., 2010). Owing to the low surface energy of PDMS and the hydrophobicity of SiO_2_ nanoparticles, the final coating exhibited superhydrophobicity with water contact angles (WCA) higher than 150° (Figure 5B).

Similar coating methods have been studied, such as casting, spray-coating, dip-coating, and other methods (Garcia et al., 2010; Zhou et al., 2012; Li et al., 2013; Selim et al., 2018b; Elzaabalawy et al., 2019; Kamelian et al., 2019; Saadatbakhsh et al., 2020), which demonstrates the efficiency of this strategy. Superhydrophobic coatings can be obtained using PDMS and hydrophilic SiO_2_ nanoparticles (owing to the migration of PDMS molecular chains to the surface (Ju et al., 2017; Su et al., 2017; Davis et al., 2018)) and using PDMS and other types of nanoparticles, such as carbon black (Zhai et al., 2019), TiO_2_ (Qing et al., 2019; Chen et al., 2022), flame soot (Shen et al., 2013), and other materials (Su et al., 2018; Wang et al., 2018; Li et al., 2022a; Pakdel et al., 2022). Additionally, other functionalities can be combined with superhydrophobicity. For example, Esfandiar Pakdel et al. reported that coatings of PDMS and natural yak hair melanin particles prepared *via* a dip–pad–dry–cure process exhibited superhydrophobicity, UV protection, and personal thermal management properties (Pakdel et al., 2022).

**FIGURE 6:**
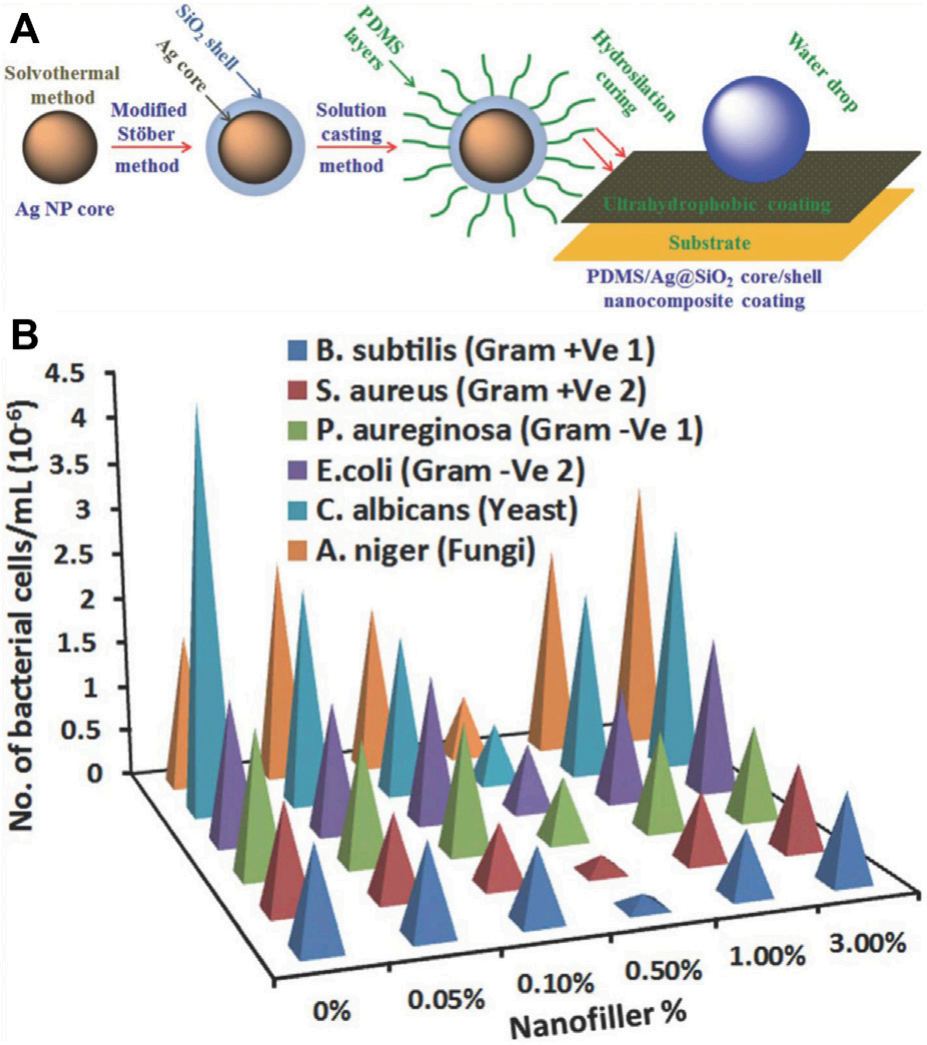
Superhydrophobic coatings fabricated by PDMS and Ag@SiO_2_ core–shell composite zero-dimensional nanoparticles **(A)**. Antifouling behavior of bacteria, yeast, and fungi strains on PDMS materials with different contents of Ag@SiO_2_ nanoparticles **(B)**. Reprinted with permission from Ref. (Selim et al., 2018a).

In addition to single-material spherical nanoparticles, there is another type of zero-dimensional nanoparticles consisting of two or more materials and denominated composite zero-dimensional nanoparticles. Their typical morphologies are core–shell structures (Selim et al., 2018a). Yong Huang et al. fabricated Ag@SiO_2_ core–shell composite zero-dimensional nanoparticles *via* a modified Stöber method and obtained a superhydrophobic PDMS and Ag@SiO_2_ coating *via* a solution casting method (Figure 6A) (Selim et al., 2018a). The superhydrophobic coating exhibited excellent antifouling abilities against various bio-foulants (Figure 6B).

### Polydimethylsiloxane with one-dimensional nanoparticles

**FIGURE 7:**
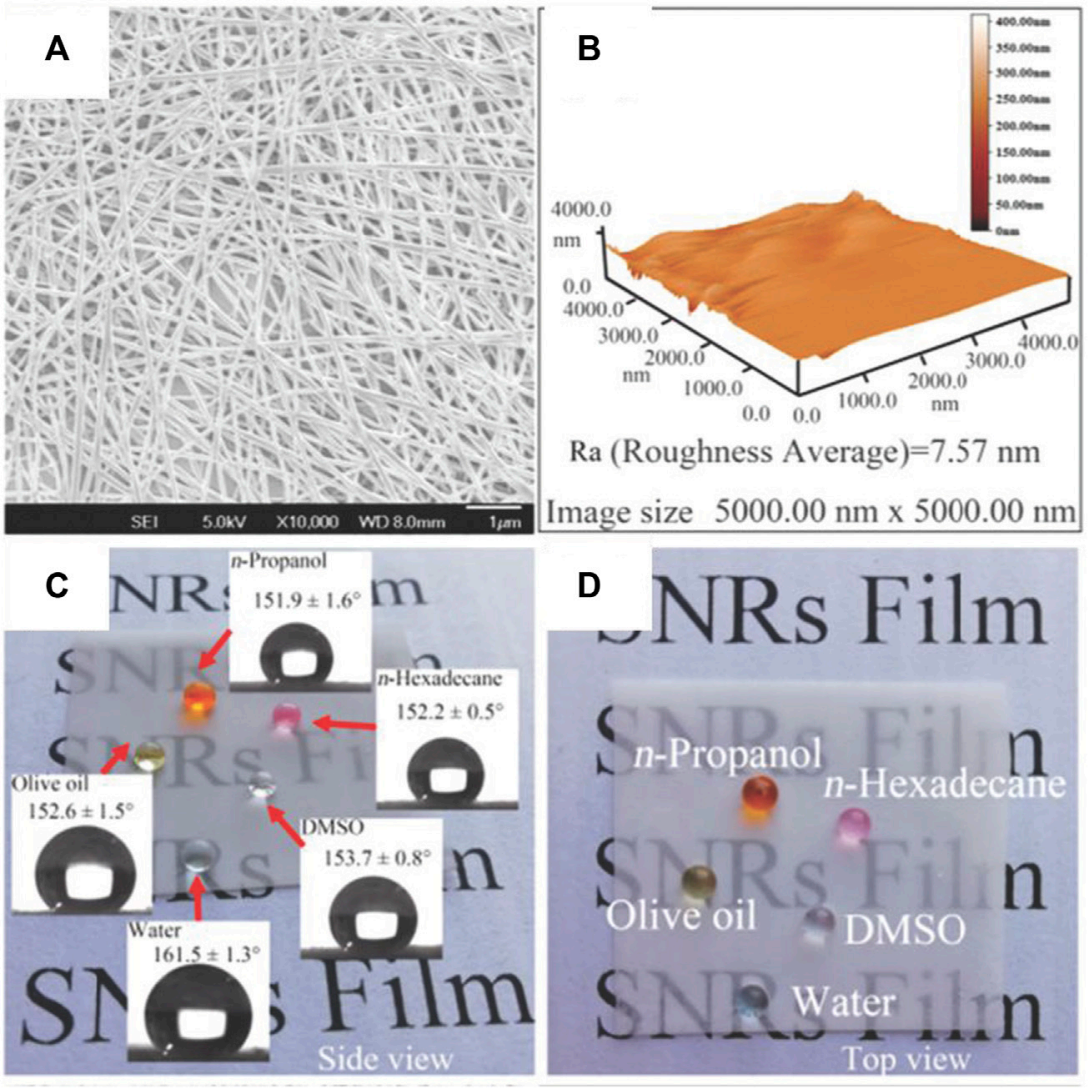
SEM **(A)**, atomic force microscopy **(B)** images, and antiwetting behavior **(C,D)** of a PDMS and Ag nanowire membrane. Reprinted with permission from Ref. (Li et al., 2020). Copyright 2020, American Chemical Society.

One-dimensional nanoparticles can exhibit nanorod (Selim et al., 2019; Selim et al., 2022a; Selim et al., 2022b), nanowire (Zhang et al., 2013; Li et al., 2020), nanofilament (Zhou et al., 2022), nanotube (Wang et al., 2010), and nanofiber structures (Chen et al., 2009; Liang et al., 2020), among other morphologies. Mohamed S. Selim et al. fabricated a superhydrophobic nanocoating based on PDMS and ZnO nanorods; the coating exhibited long-term antifouling abilities for marine applications (Selim et al., 2019). Zhiguang Guo et al. performed spray-coating of a silk fibroin membrane using a mixture of PDMS and Ag nanowires (Figure 7) (Li et al., 2020). SEM and atomic force microscopy images shown in Figures 7A and 7B revealed the hierarchical structures of a PDMS and Ag nanowire membrane. The prepared membranes exhibited superhydrophobicity, self-cleaning, and antifouling properties (Figures 7C and 7D). Superhydrophobic coatings with similar morphologies have also been obtained *via* a simple coating process with PDMS and TiO_2_ nanowires (Zhang et al., 2013) or CNTs (Wang et al., 2010).

### Polydimethylsiloxane with two-dimensional nanoparticles

**FIGURE 8:**
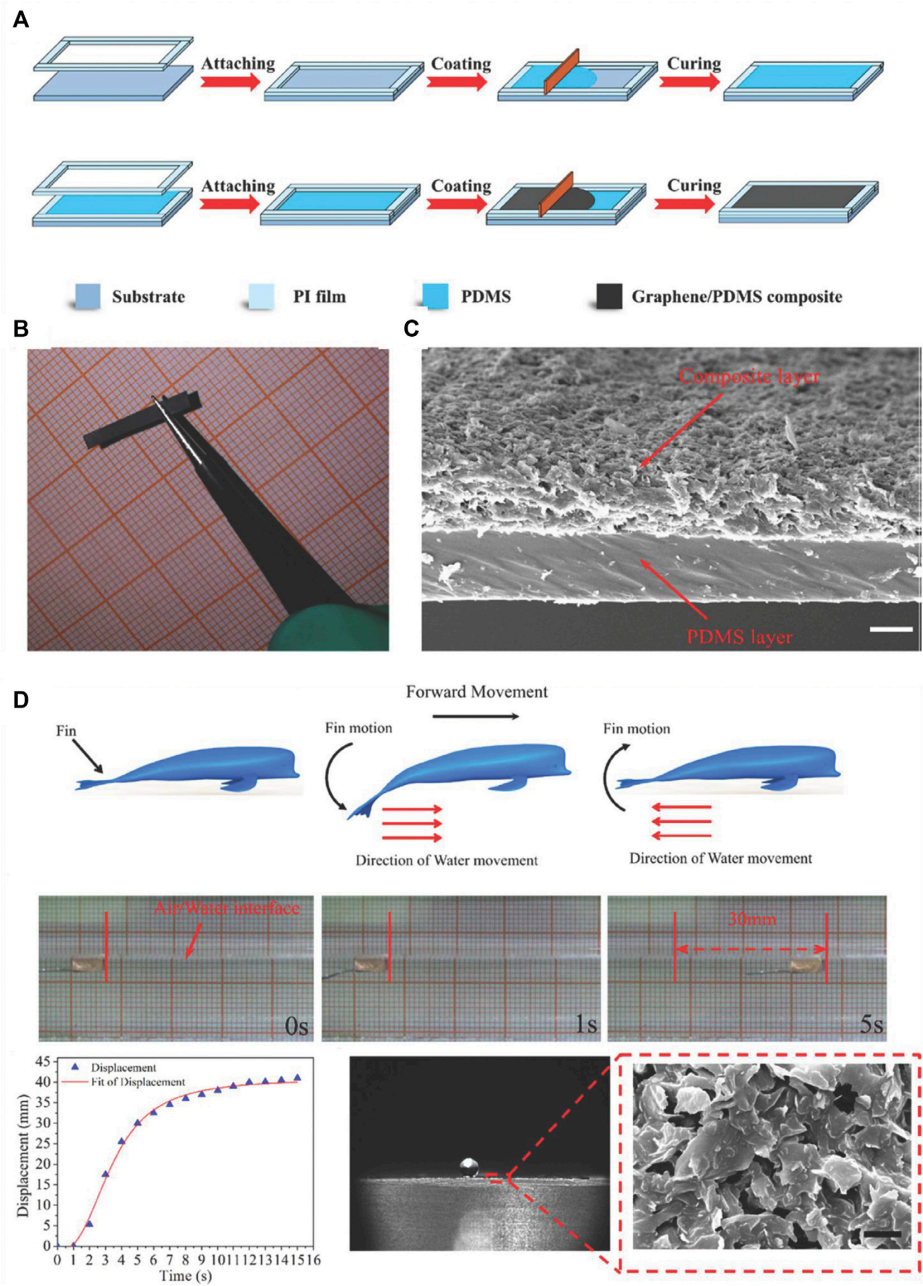
Fabrication process of PDMS and graphene materials **(A)**. Optical **(B)** and SEM **(C)** images of PDMS and graphene materials. Photo-responsive and superhydrophobic properties of PDMS and graphene materials **(D)**. Reprinted with permission from Ref. (Wang et al., 2019b). Copyright 2019, American Chemical Society.

Two-dimensional nanoparticles can be made of materials such as montmorillonite (Song et al., 2015; Kancı Bozoğlan et al., 2021; Peng et al., 2021), layered silicates (Ho et al., 2012), mica (Han et al., 2019; Miyamoto et al., 2019), graphite (Li and Guo, 2020), graphene, and GO (Wang et al., 2019b; Saharudin et al., 2019; Wang et al., 2021d; Selim et al., 2022a; Selim et al., 2022b). Here, we use the emerging graphene materials as an example to introduce the fabrication of superhydrophobic materials made with PDMS with two-dimensional nanoparticles. Owing to the multi-functionality of graphene, excellent superhydrophobic and photo-responsive properties can be achieved by pouring PDMS and graphene mixtures into templates (Figure 8A) (Wang et al., 2019b). The surface morphologies of PDMS and graphene composites are determined by the hierarchical structures of the two-dimensional graphene nanoparticles (Figures 8B and 8C). The displacement of the beluga whale robot is shown in Figure 8D, and this phenomenon is attributed to the superhydrophobicity of PDMS and graphene composites.

### Polydimethylsiloxane with three-dimensional nanoparticles

**FIGURE 9:**
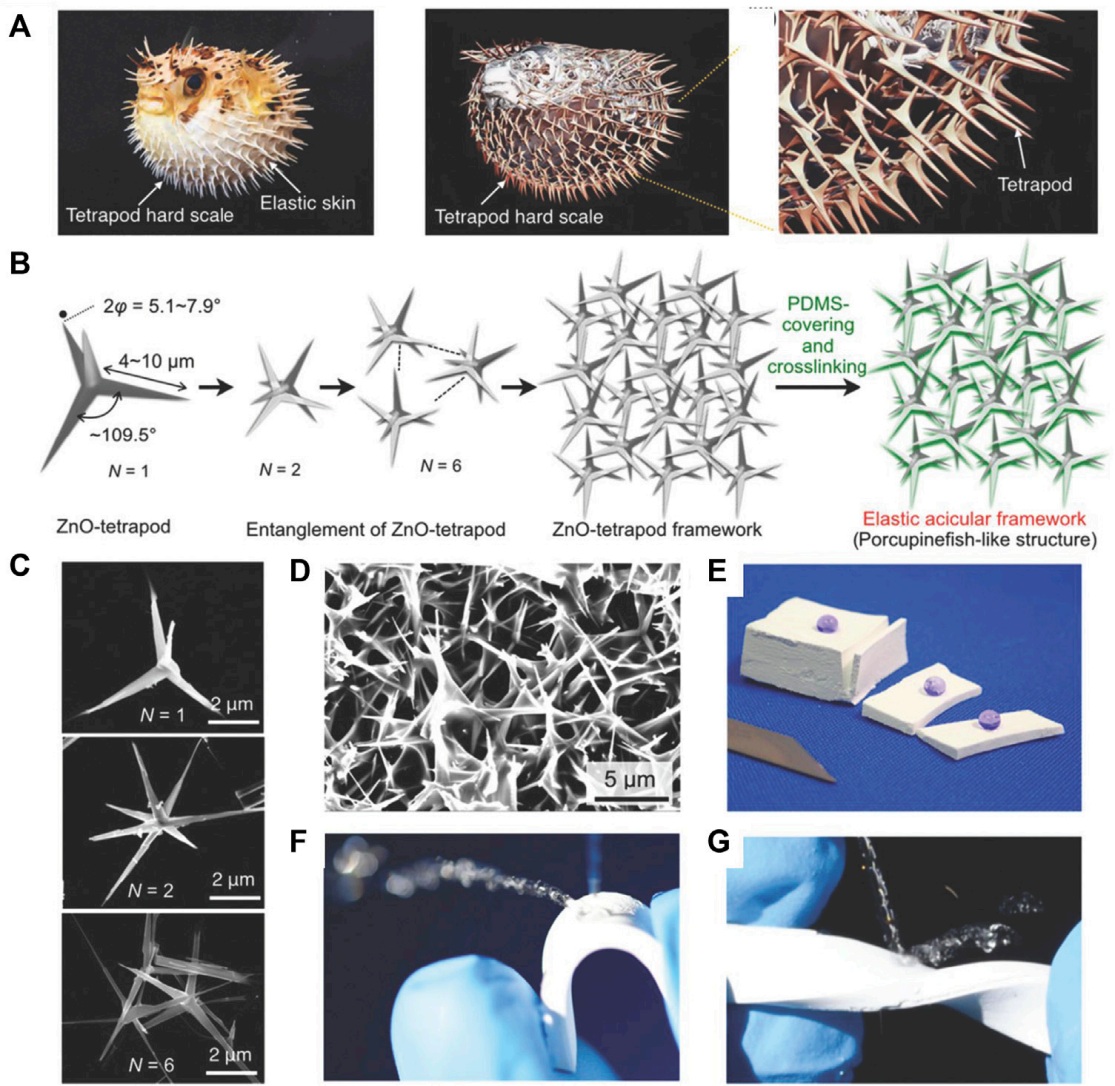
Photograph and computer tomography scan images of a porcupinefish and its skeleton **(A)**. Schematic representation of independent tetrapod-shaped ZnO and its composite with PDMS **(B)**. SEM images of tetrapod-shaped ZnO **(C)** and elastic acicular frameworks **(D)**. Photographs of the materials showing superhydrophobicity with slicing resistance **(E)**, bending resistance **(F)**, and twisting resistance **(G)**. Reprinted with permission from Ref. (Yamauchi et al., 2019). Copyright 2019, American Chemical Society.

Three-dimensional nanoparticles can be categorized into two types. The first type are particles made of single materials, such as tetrapod-shaped ZnO (Yamauchi et al., 2019) and flower-like CaTiO_3_ structures (Wang et al., 2007). As shown in Figures 9A–9D, Yoshihiro Yamauchi et al. reported superhydrophobic materials made of PDMS and tetrapod-shaped ZnO with porcupinefish-like structures obtained by pouring the composite into a template (Yamauchi et al., 2019). The composite materials exhibited superhydrophobicity not only on the surface but also inside; thus, the superhydrophobicity can be stable even under material abrasion, bending, or twisting deformation (Figures 9E–9G).

**FIGURE 10:**
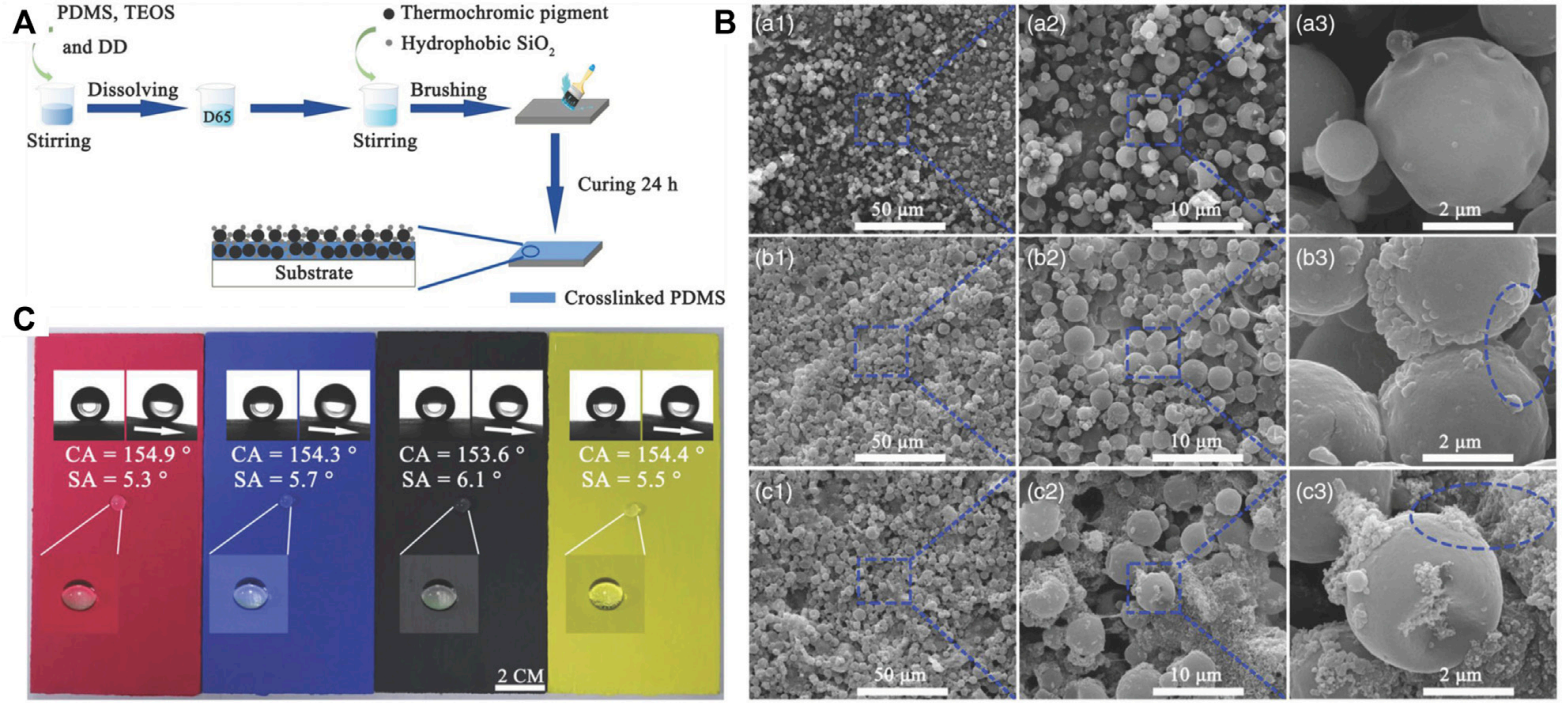
Schematic diagram of the fabrication process of thermochromic superhydrophobic coatings **(A)**. SEM images of a blue TP powder and various coatings **(B)**: blue TP powder (a1–a3); blue TP/coating without SiO_2_ (b1–b3); blue TP/coating containing SiO_2_ (c1–c3). Surface wettability measurements for different coatings **(C)**: the red, blue, black, and yellow TP/coating. Reprinted with permission from Ref. (Cheng et al., 2022). Copyright 2021, Wiley-VCH GmbH.

The second type of three-dimensional nanoparticles are those consisting of a combination of two or more materials. As shown in Figure 10, three-dimensional nanoparticles with a raspberry-like morphology have been obtained *via* the aggregation of spherical SiO_2_ nanoparticles on the surface of spherical thermochromic pigment (TP) particles (Cheng et al., 2022). A thermochromic superhydrophobic coating has been fabricated by brushing a mixture of PDMS and the three-dimensional nanoparticles onto a substrate (Figure 10A). SEM images of a blue TP powder and various coatings are shown in Figure 10B. Coatings made with different TP particles show similar superhydrophobicity, and surface contaminants can easily be washed with water (Figure 10C). A similar superhydrophobic surface made of nanoparticles with zero-dimensional morphology and different sizes was obtained by combining PDMS and CaCO_3_/SiO_2_ composite particles with a raspberry-like morphology (Yang et al., 2009). Sumit Barthwal et al. reported a stable superhydrophobic coating based on PDMS and three-dimensional composite nanoparticles assembled with one-dimensional multi-walled CNTs and ZnO nanorods (Barthwal et al., 2020). The multi-walled CNT and ZnO composite nanoparticles were prepared *via* a sol-gel method, and superhydrophobic coatings were obtained by dip-coating various substrates.

**FIGURE 11:**
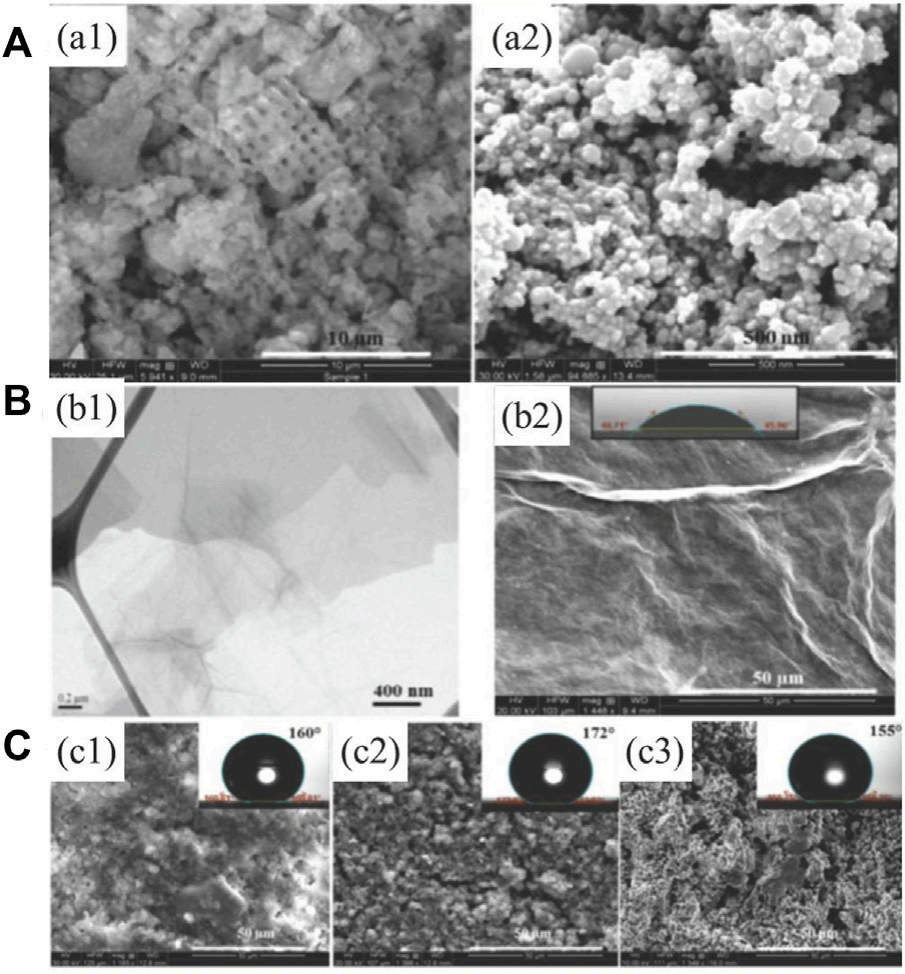
SEM images of DE and TiO_2_ (P25) nanoparticles **(A)**. TEM image of exfoliated GO and SEM image of dried GO flakes **(B)**. SEM images and WCAs on superhydrophobic coating fabricated with PDMS and DE, DE/TiO_2_, or DE/TiO_2_/rGO particles **(C)**. Reprinted with permission from Ref. (Nine et al., 2015). Copyright 2015, American Chemical Society.

Three-dimensional composite nanoparticles can also consist of combinations of nanoparticles with different dimensional morphologies. Mohamed S. Selim et al. developed a simple two-phase process to obtain three-dimensional composite nanoparticles with one-dimensional anatase TiO_2_ nanorods (Selim et al., 2022a) or boehmite nanorods (c-AlOOH) (Selim et al., 2022b) on the surface of two-dimensional GO sheets. PDMS and three-dimensional composite nanoparticles consisting of nanorods on the surface of GO sheets can be coated onto substrates such as a hull to confer superhydrophobicity and antifouling abilities to the surface. Dusan Losic et al. prepared graphene-based superhydrophobic composite coatings with diatomaceous earth (DE), reduced GO (rGO) and TiO_2_ (P25) nanoparticles *via* spraying, brush painting, and dip coating (Nine et al., 2015). The morphologies of DE, TiO_2_ (P25) and GO particles can be found in Figures 11A and 11B. Due to the hydrophilicity of GO (Figure 11B, WCA 45°), the final superhydrophobic coating was prepared with rGO to avoid the effect of hydrophilic GO. Finally, the coatings with PDMS and DE, DE/TiO_2_, or DE/TiO_2_/rGO composite particles showed rough surface morphologies and superhydrophobicity (Figure 11C).

Owing to the wide variety of potential morphologies of three-dimensional composite nanoparticles, it is impossible to discuss them thoroughly in this review. Nonetheless, other morphologies of three-dimensional composite nanoparticles can be reasonably conceived based on the “3M” methodology and the previous discussion (Dai et al., 2021; Li et al., 2022b; Li et al., 2022c). For example, zero-dimensional carbon black nanoparticles and one-dimensional carbon nanotubes have been mixed to obtain conductive composite nanoparticles, and superhydrophobic materials could be obtained by mixing PDMS and the conductive composite nanoparticles in a solution and curing the PDMS (Li et al., 2022b).

## Methods to obtain superhydrophobicity based on polydimethylsiloxane and nanoparticles

**FIGURE 12:**
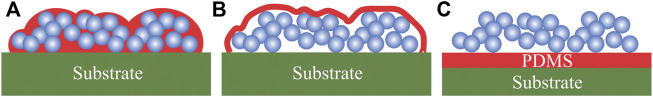
Strategies to obtain superhydrophobicity using PDMS and nanoparticles. Mixing PDMS and nanoparticle aggregates **(A)**; PDMS on the surface of the nanoparticle aggregates **(B)**; Nanoparticle aggregates on a PDMS surface **(C)**.

The “3M” methodology can be applied to prepare a variety of superhydrophobic coatings. In addition to the previous examples, in which superhydrophobic materials for antifouling applications were obtained based on a combination of PDMS with nanoparticle aggregates (shown for zero-dimensional nanoparticles as an example in Figure 12A), two other strategies can be used to obtain superhydrophobic materials using PDMS and nanoparticles (Figures 12B and 12C).

**FIGURE 13:**
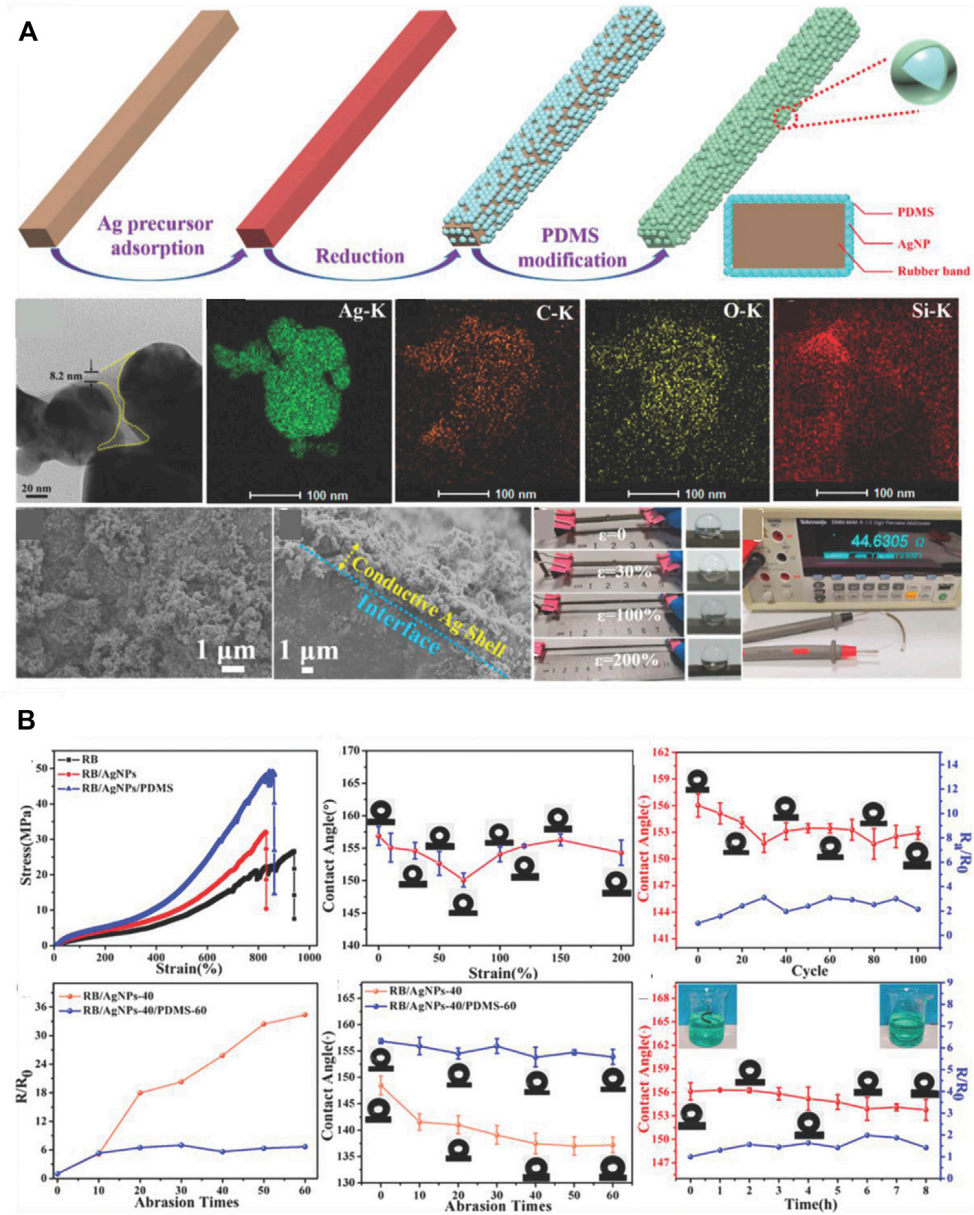
Preparation of superhydrophobic composites PDMS on the surface of AgNPs **(A)**. Durable superhydrophobicity of the composites under cyclic stretching–releasing and abrasion tests **(B)**. Reprinted with permission from Ref. (Wang et al., 2019c). Copyright 2019, American Chemical Society.

When PDMS is added on the surface of nanoparticle aggregates (Figure 12B), the surface of the final superhydrophobic material consists of PDMS. Therefore, hydrophilic particles could be used without hydrophobic modifications because the PDMS provides the required hydrophobicity. Figure 13A shows an example in which Ag nanoparticles (AgNPs) were first applied on a rubber band (RB) substrate, and PDMS was then coated on the substrate with AgNPs to confer superhydrophobicity to the material (Wang et al., 2019c). Owing to the existence of a continuous PDMS film on the surface, the superhydrophobicity can be maintained under cyclic stretching–releasing and abrasion tests (Figure 13B).

**FIGURE 14:**
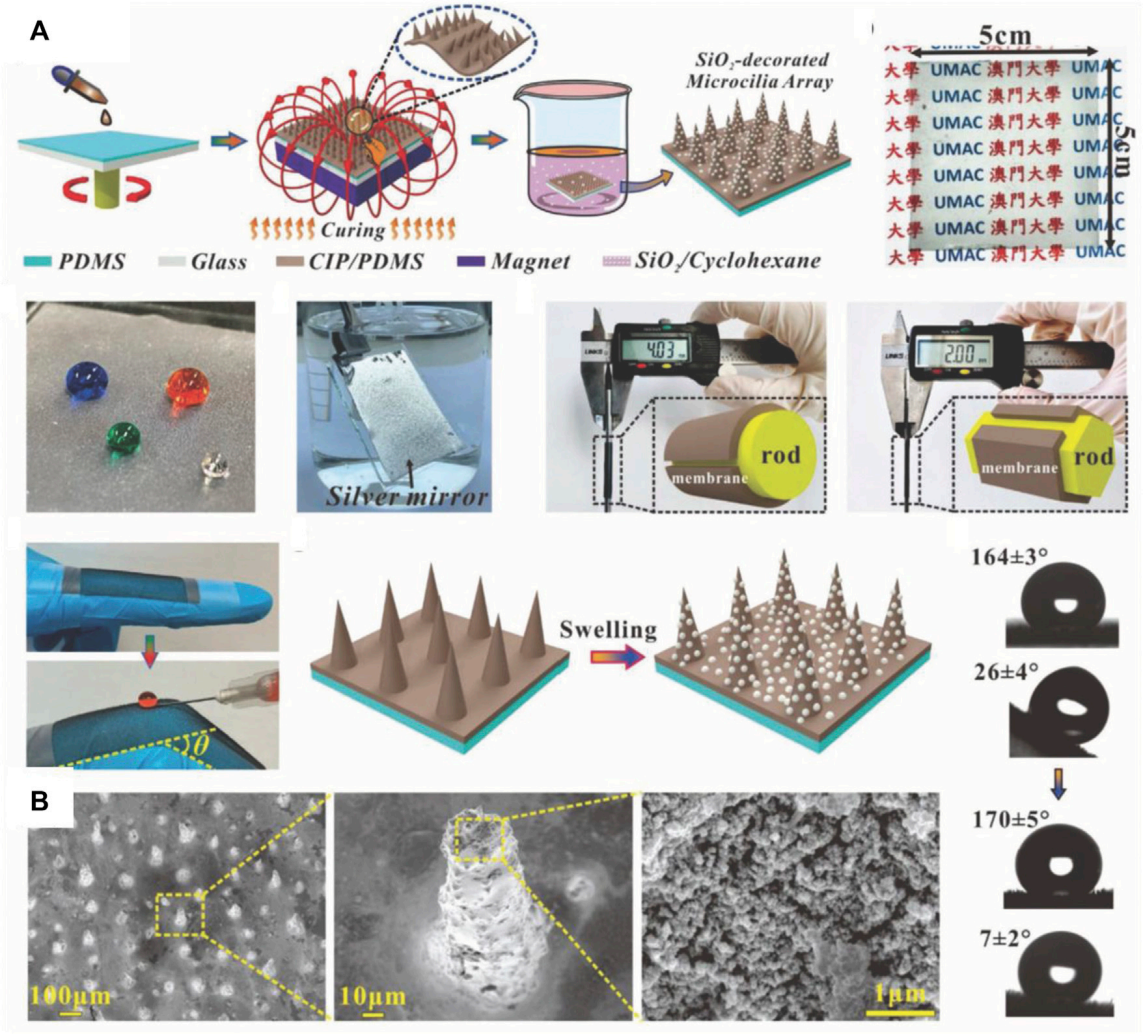
Preparation of superhydrophobic coatings by adding hydrophobized SiO_2_ nanoparticles on a PDMS and CIP microcilia array **(A)**. SEM images of the surfaces **(B)**. Reprinted with permission from Ref. (Dai et al., 2021). Copyright 2020, Wiley-VCH GmbH.

When nanoparticle aggregates are added on a PDMS surface (Figure 12C), the surface of the final superhydrophobic material consists of nanoparticles. Therefore, the nanoparticles must be hydrophobic to avoid conferring a hydrophilic or superhydrophilic character to the surface. Figure 14A shows an example in which hydrophilic SiO_2_ nanoparticles hydrophobized with PDMS are closely laid on a PDMS and carbonyl iron particle (CIP) microcilia array to form a superhydrophobic coating (Dai et al., 2021). The nanoparticle aggregates provide the necessary roughness to achieve superhydrophobicity (Figure 14B). For this type of superhydrophobic materials, the substrate may be a thick PDMS layer, which would expand the applications of PDMS-based superhydrophobic materials (Figure 14A).

## Conclusion and outlook

In this review, according to the mechanism of superhydrophobicity based on dual micro-scale and nano-scale structures, or hierarchical roughness, we propose a versatile “3M” methodology (materials, methods, and morphologies) that can be defined as “The use of specific materials and methods to construct special morphologies for surface superhydrophobicity” to guide the fabrication of superhydrophobic PDMS-materials for antifouling applications. Three types of PDMS-based materials were introduced: pure PDMS materials, materials consisting of PDMS and nanoparticles, and combinations of PDMS and other materials. Furthermore, the methods that can be chosen were discussed based on the different types of materials. Because materials made of PDMS and nanoparticles are advantageous, they were discussed to exemplify various morphologies and explain the “3M” methodology to obtain superhydrophobicity. Owing to the wide variety of potential morphologies of zero-, one-, two-, and three-dimensional nanoparticles, it is impossible to discuss them thoroughly in this review. Nonetheless, typical materials, methods, and morphologies were carefully selected and reviewed. Based on this “3M” methodology, in future research, people can design various novel morphologies, and obtain necessary dual micro-scale and nano-scale structures, or hierarchical roughness by adopting novel materials or methods. Therefore, numerous novel superhydrophobic materials will be explored. This paper is expected to serve as a helpful reference to future research on the fabrication of superhydrophobic materials based on PDMS and other polymers for antifouling applications. Moreover, superhydrophobic antifouling materials with multifunctions, such as optical, electrical, magnetic, thermo function, will have extensive applications in biomedical devices, lab-on-a-chip devices, sensors, etc. The challenges in future research should be focused on developing novel cheap and safe raw materials, versatile and covenitent fabrication methods, and designable but easily achieveable and stable enough morphologies.
