# Supplementary material for: A versatile “3M” methodology to obtain superhydrophobic PDMS-based materials for antifouling applications
Source: Front Bioeng Biotechnol. 2022 Aug 29;10:998852. doi: 10.3389/fbioe.2022.998852 (PMC9464926; doi:10.3389/fbioe.2022.998852)
Supplement: Supplementary file 1 [file Presentation1.pdf]

A versatile "3M" methodology to obtain superhydrophobic PDMS-based materials for antifouling applications

Zhoukun He[1], Xiaochen Yang[1, 2], Linpeng Mu[1, 2], Na Wang[1, 2], Xiaorong Lan [3, 4*]

[1]Institute for Advanced Study, Research Center of Composites and Surface and Interface Engineering, Chengdu University, Chengdu, China, [2]School of Mechanical Engineering, Chengdu University, Chengdu, China, [3]Luzhou Key Laboratory of Oral & Maxillofacial Reconstruction and Regeneration, The Affiliated Stomatological Hospital of Southwest Medical University, Luzhou, China, [4]Institute of Stomatology, Southwest Medical University, Luzhou, China.

*. Corresponding author

Xiaorong Lan: xiaoronglancdm@163.com

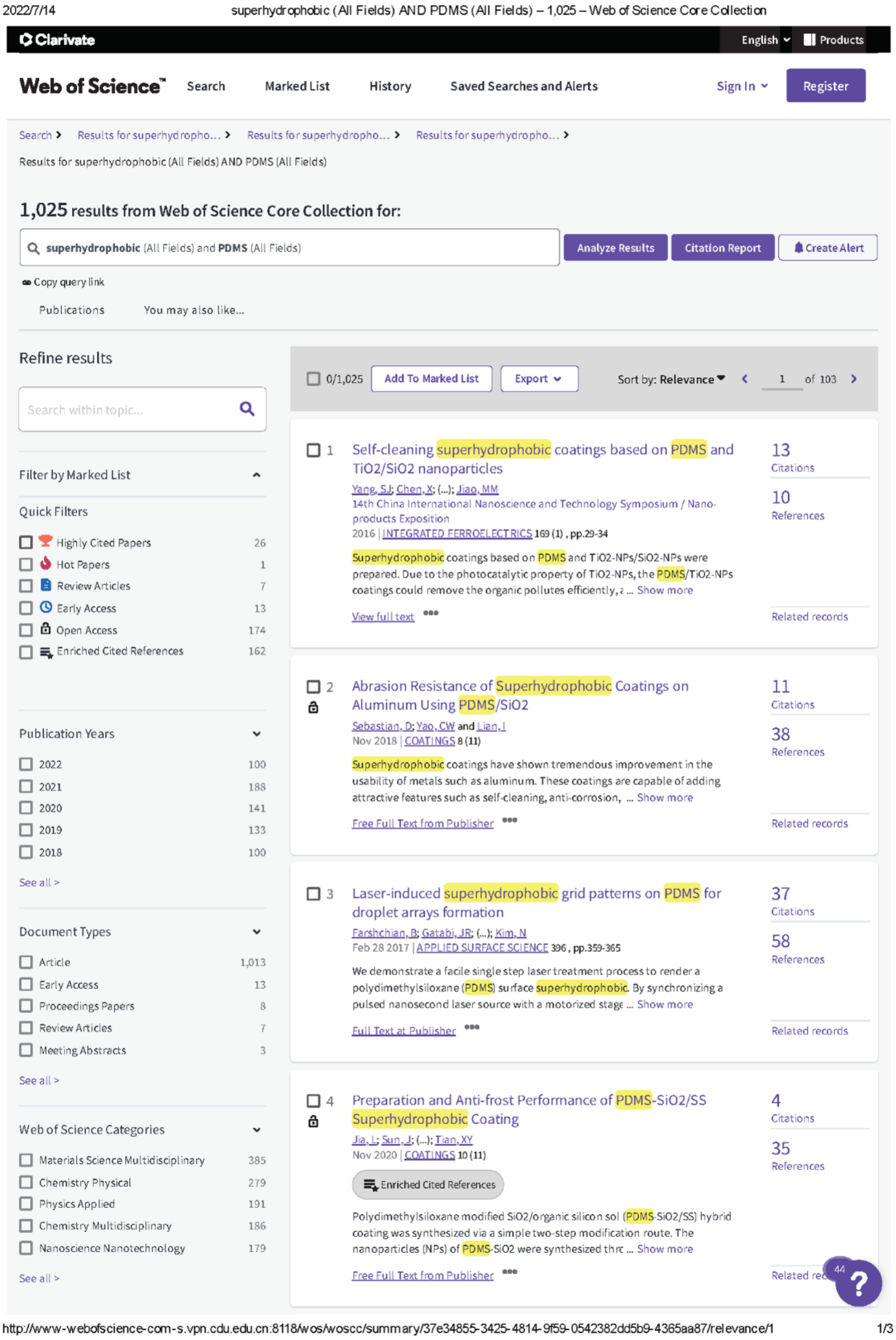

Fig. S1. 1,025 publications searched in all fields in Web of Science with “superhydrophobic” and “PDMS” on July 14th, 2022.

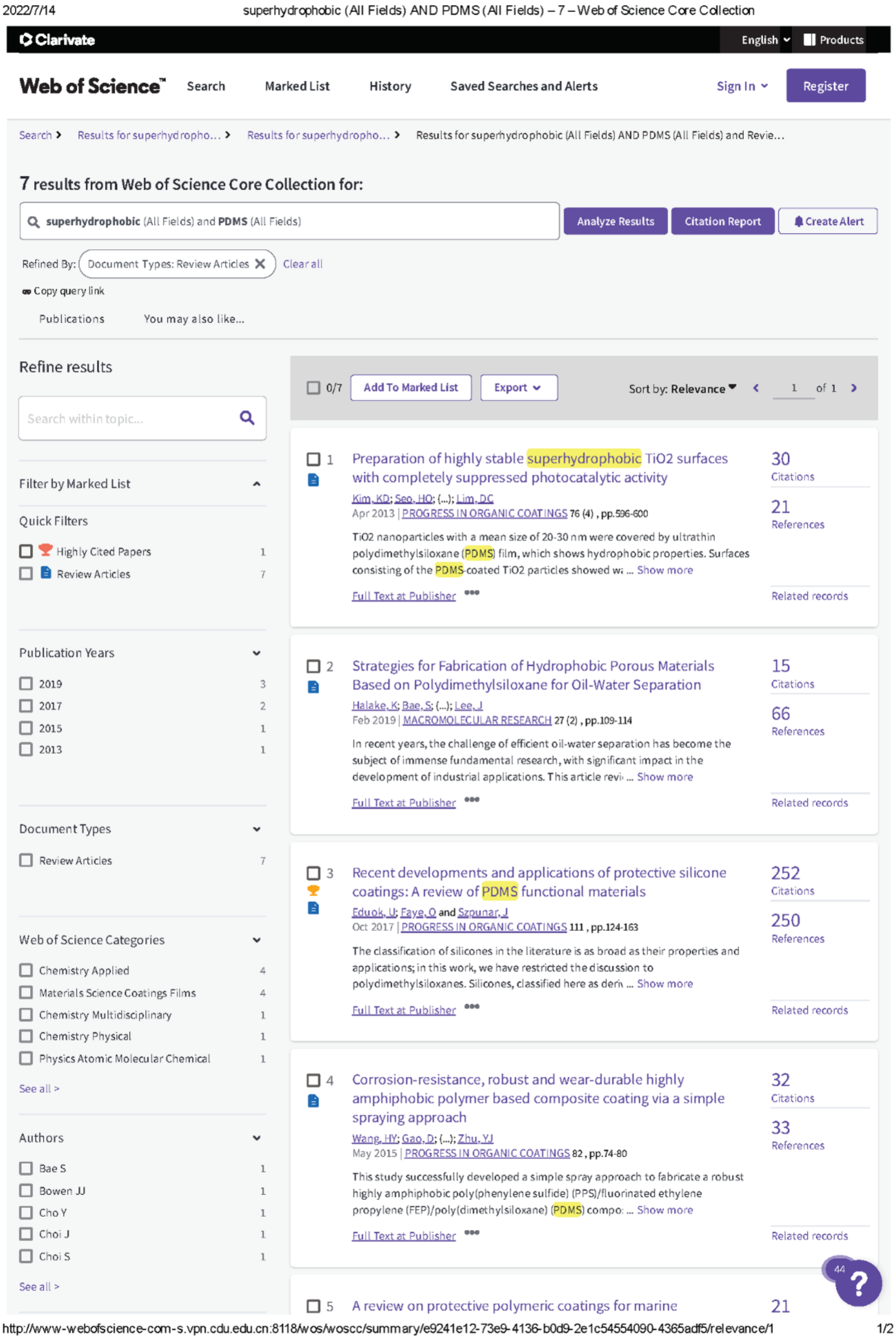
2022/7/14 superhydrophobic (All Fields) AND PDMS (All Fields) – 7 – Web of Science Core Collection

Clarivate English Products

Web of Science™ Search Marked List History Saved Searches and Alerts Sign In Register

Search > Results for superhydropho... > Results for superhydropho... > Results for superhydrophobic (All Fields) AND PDMS (All Fields) and Revie...

7 results from Web of Science Core Collection for:

superhydrophobic (All Fields) and PDMS (All Fields)

Analyze Results | Citation Report | Create Alert

Refined By: Document Types: Review Articles × Clear all

Copy query link

Publications You may also like...

Refine results

Search within topic...

Filter by Marked List

Quick Filters

- Highly Cited Papers 1
- Review Articles 7

Publication Years

- 2019 3
- 2017 2
- 2015 1
- 2013 1

Document Types

- Review Articles 7

Web of Science Categories

- Chemistry Applied 4
- Materials Science Coatings Films 4
- Chemistry Multidisciplinary 1
- Chemistry Physical 1
- Physics Atomic Molecular Chemical 1

See all >

Authors

- Bae S 1
- Bowen JJ 1
- Cho Y 1
- Choi J 1
- Choi S 1

See all >

0/7 Add To Marked List Export Sort by: Relevance 1 of 1

1 Preparation of highly stable superhydrophobic TiO2 surfaces with completely suppressed photocatalytic activity — 30 Citations, 21 References

Kim, KD; Seo, HO; (...); Lim, DC

Apr 2013 | PROGRESS IN ORGANIC COATINGS 76 (4), pp.596-600

TiO2 nanoparticles with a mean size of 20-30 nm were covered by ultrathin polydimethylsiloxane (PDMS) film, which shows hydrophobic properties. Surfaces consisting of the PDMS-coated TiO2 particles showed w... Show more

Full Text at Publisher ••• Related records

2 Strategies for Fabrication of Hydrophobic Porous Materials Based on Polydimethylsiloxane for Oil-Water Separation — 15 Citations, 66 References

Halake, K; Bae, S; (...); Lee, J

Feb 2019 | MACROMOLECULAR RESEARCH 27 (2), pp.109-114

In recent years, the challenge of efficient oil-water separation has become the subject of immense fundamental research, with significant impact in the development of industrial applications. This article revi... Show more

Full Text at Publisher ••• Related records

3 Recent developments and applications of protective silicone coatings: A review of PDMS functional materials — 252 Citations, 250 References

Eduok, U; Faye, O and Szpunar, J

Oct 2017 | PROGRESS IN ORGANIC COATINGS 111, pp.124-163

The classification of silicones in the literature is as broad as their properties and applications; in this work, we have restricted the discussion to polydimethylsiloxanes. Silicones, classified here as deri... Show more

Full Text at Publisher ••• Related records

4 Corrosion-resistance, robust and wear-durable highly amphiphobic polymer based composite coating via a simple spraying approach — 32 Citations, 33 References

Wang, HY; Gao, D; (...); Zhu, YJ

May 2015 | PROGRESS IN ORGANIC COATINGS 82, pp.74-80

This study successfully developed a simple spray approach to fabricate a robust highly amphiphobic poly(phenylene sulfide) (PPS)/fluorinated ethylene propylene (FEP)/poly(dimethylsiloxane) (PDMS) compo... Show more

Full Text at Publisher ••• Related records

5 A review on protective polymeric coatings for marine — 21

http://www-webofscience-com-s.vpn.cdu.edu.cn:8118/wos/woscc/summary/e9241e12-73e9-4136-b0d9-2e1c54554090-4365adf5/relevance/1 1/2

Fig. S2. 7 review publications searched in all fields in Web of Science with "superhydrophobic" and "PDMS" on July 14th, 2022.
